# Rubber Antioxidants and Their Transformation Products: Environmental Occurrence and Potential Impact

**DOI:** 10.3390/ijerph192114595

**Published:** 2022-11-07

**Authors:** Jing Xu, Yanfen Hao, Zhiruo Yang, Wenjuan Li, Wenjing Xie, Yani Huang, Deliang Wang, Yuqing He, Yong Liang, Julius Matsiko, Pu Wang

**Affiliations:** 1 State Key Laboratory of Precision Blasting, Jianghan University, Wuhan 430056, China; 2 Hubei Key Laboratory of Environmental and Health Effects of Persistent Toxic Substances, School of Environment and Health, Jianghan University, Wuhan 430056, China; 3 Department of Chemistry, Faculty of Science, Muni University, Arua P.O. Box 725, Uganda

**Keywords:** rubber antioxidants, transformation products, toxic effects, occurrence, environmental impacts

## Abstract

Antioxidants are prevalently used during rubber production to improve rubber performance, delay aging, and extend service life. However, recent studies have revealed that their transformation products (TPs) could adversely affect environmental organisms and even lead to environmental events, which led to great public concern about environmental occurrence and potential impacts of rubber antioxidants and their TPs. In this review, we first summarize the category and application of rubber antioxidants in the world, and then demonstrate the formation mechanism of their TPs in the environment, emphasizing their influence on the ozone oxidative degradation. The potential toxic effects of antioxidants and their TPs are further reviewed to improve understanding of their biological health impact and environmental risks. Finally, the environmental occurrences of antioxidants and their TPs are summarized and their environmental impacts are demonstrated based on the recent studies. Due to the currently limited understanding on the toxic and biological effects of these compounds, further studies are required in order to better assess various TPs of these antioxidants and their environmental impact. To our knowledge, this is the first review on antioxidants and their TPs in the environment, which may elevate the environmental risk awareness of rubber products and their TPs in the near future.

## 1. Introduction

Many unidentified chemicals and related transformation products (TPs) are released into the environment during the life-cycle of commercial products. Efforts in evaluating their adverse impacts were usually not taken until these chemicals become globally ubiquitous. Rubber is a group of high-molecular-weight polymer materials with a property of elasticity at 20–27 °C [1], and rubber is one of such typical commercial products. Rubber products are mainly used in industrial and agricultural production, transportation, and national defense construction, and antioxidants are a group of the most important chemicals with widespread use in rubber products. Due to exposure to ozone (O_3_) (O_3_ oxidative degradation), light (photo-degradation), heat (thermo-degradation), redox processes, catalysis of heavy metals (e.g., copper) [2], radiation, and erosion of other chemicals and molds (bio-degradation), rubber products may become sticky, hard, brittle, or cracked after long-term use or storage [3]. O_3_ oxidative degradation is the most common pathway causing aging because of the strong oxidation effect of superoxide anion radical (O_2_^•−^) produced by O_3_. Rubber aging leads to a gradual reduction in its performance and even total loss of its use value, which paves the way for the addition of antioxidants in rubber. Antioxidants are added to natural rubber (NR) and synthetic rubber (SR) during mastication, which is the process of transforming rubber from a strong and elastic state to a soft and plastic state [4,5]. In addition, they could also be coated on the NR surface to achieve a similar effect [6]. In spite of this, the antioxidants may still be transformed in the environment due to oxidative degradation and may produce some novel compounds [7].

Given the global ubiquity of the antioxidants, the potential adverse impact of these chemicals and their TPs has gradually caused public concern. Many previous studies have noticed the degradation of antioxidants through the O_3_ oxidation, since the most observed TPs of antioxidants contains the quinone group and they showed high toxicity. Antioxidants and their TPs have been released into the environment, and may lead to adverse impacts on the local biota and even human health. Recently, it was reported that the rubber antioxidant *N*-(1,3-dimethylbutyl)-*N′*-phenyl-*p*-phenylenediamine (6PPD or antioxidant 4020), a typical tire rubber antioxidant, could enter the surrounding environment together with tire-wear particles (TWPs) [7,8]. Its TP (6PPD-quinone) could induce acute mortality in coho salmon, and therefore assumed to result in a large number of silver salmon deaths in the US Pacific Northwest [7]. In addition, large amounts of antioxidants used as rubber additives in tire filtrates have been observed to substantially reduce the survival and reproduction rates of soil worms by more than 25% and 50%, respectively [9]. This implies that rubber antioxidants in tire filtrates could disturb microorganisms in the surrounding soil, reduce the number of soil worms, and even threaten the terrestrial ecosystem by affecting soil organisms and their intestinal microbiota. These studies therefore aroused great attention on the environmental impacts of rubber antioxidants and their TPs.

To date, there is no review to summarize the environmental occurrence and impact of rubber antioxidants and their TPs. In the present work, we first introduce the production and usage of antioxidants in the world, and then demonstrate the formation mechanism of some reported TPs in the environment. Their potential toxic effects are further introduced, and thereafter, the occurrence and environmental impact of rubber antioxidants and their TPs are summarized based on the previous studies. Since amine antioxidants have been widely used and their TPs have been intensively studied recently, more abundant results about them are stressed in this review, which may enhance the understanding of the environmental transformation of antioxidants and the potential environmental risks of their TPs.

## 2. Production and Use of Typical Rubber Antioxidants

Rubber antioxidants are defined as substances that could delay the aging of polymer compounds and prolong the service life of rubber products by inhibiting oxidation, heat, or light radiation [10]. To date, the annual global consumption of rubber antioxidants is over 700,000 tons, accounting for about 40% of the total amount of rubber additives. This is about twice higher than that of phosphorus flame retardants, a group of emerging pollutants which received great attention in the past decades [11]. China is one of the main countries producing rubber antioxidants, and the production accounts for more than 70% of the total amount globally. The production of rubber antioxidants in China ranged from 365,000 to 378,000 tons during 2016–2020, showing a constant annual trend [12]. Amine antioxidants are the main rubber antioxidants produced and used in China, of which 6PPD and 2,2,4-Trimethyl-1,2-dihydroquinoline (TMQ, RD) have the highest production, accounting for more than 80% of the total amine antioxidants. Moreover, the annual production of 6PPD is 189,500–208,600 tons, which accounts for about 55% of the total amount of amine antioxidants, followed by TMQ with an annual production of 102,800–126,000 tons (approximately 30% of the total amount) [13]. There is very little information about the exact production of the other types of rubber antioxidants in the world.

**Figure 1:**
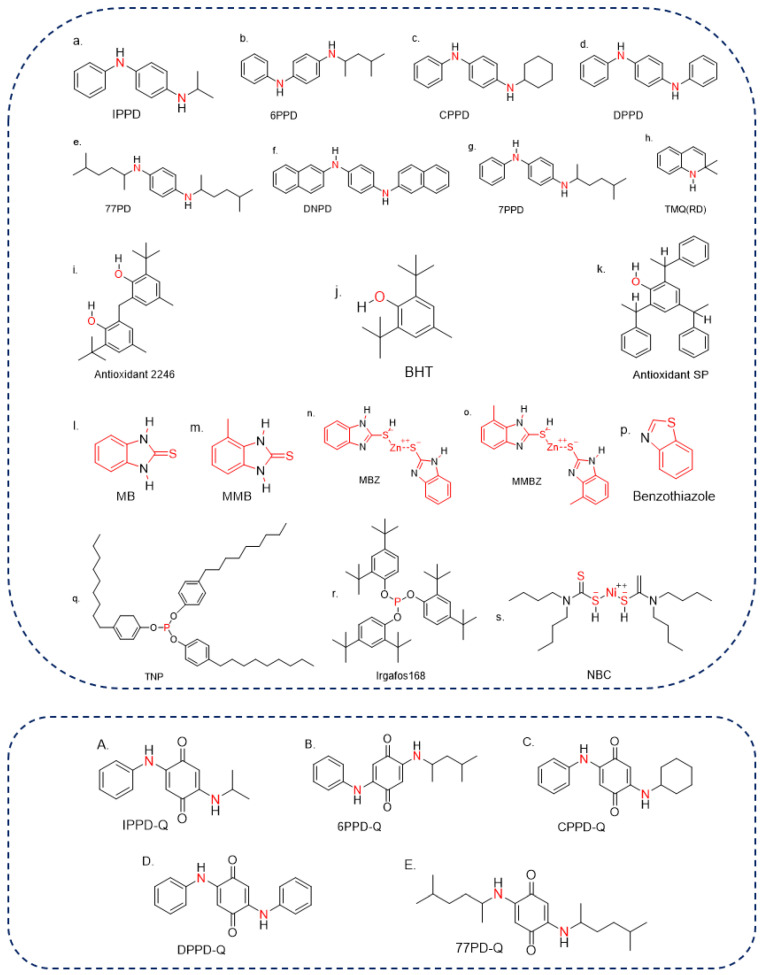
The structures of typical antioxidants and some of their TPs ((**a**–**h**) are for amine antioxidants, (**i**–**k**) are for phenolic antioxidants, (**l**–**p**) are for heterocyclic antioxidants, (**q**,**r**) are for phosphite antioxidants, (**s**) is for nickel salts antioxidants, (**A**–**E**) are for TPs, the red parts represent the similar structure of the same type of antioxidants).

Antioxidants can be classified according to their antiaging mechanisms (counteracting oxygen, O_3_, and copper), effects on appearance (discoloration vs. non-discoloration, and contamination vs. non-contamination), specific function (heat resistance, bending, and crack resistance), and physicochemical properties (natural, physical, and chemical antioxidants). Natural antioxidants are only found in NR, such as amino acids, tocotrienol, and betaines [14], whereas physical and chemical antioxidants are widely used in various synthetic rubber products. The rubber-aging process comprises three stages: initiation, reaction, and termination [15,16], and the physical antioxidants are usually used to address the initiation stage of rubber aging. A film-isolating oxygen and O_3_ is formed on the surface of rubber products by directly applying or spraying, which can prevent the rubber from aging. By contrast, chemical antioxidants are usually used to address the reaction stage of rubber aging. According to the different fracture modes of the molecular chain of raw rubber materials, different chemical antioxidants are added to block the growth reaction chain during aging [15]. Chemical antioxidants are generally classified as amine, phenolic, heterocyclic, phosphite, and nickel salts (nickel dibutyl dithiocarbamate (NBC)) antioxidants according to their chemical structure (Figure 1). During the rubber production, various antioxidants are often used as a mixture to improve performance and ensure an antiaging effect.

### 2.1. Amine Antioxidants

Amine antioxidant is the most common rubber antioxidant, which was produced as early as the 1970s and widely used in the rubber industry. Typical amine antioxidants include diaryl-secondary amine, acetone-amine condensation product, *p*-phenylenediamine, and aldehyde-amine condensation product antioxidants [17]. The most common products include 6PPD, *N,N′*-bis(1,4-dimethylpentyl)-*p*-phenylenediamine (77PD), tris-(*N*-dimethylpentyl-*p*-phenylenediamine)-*N,N′,N″*-1,3,5-triazine (PPDTZ), 1,3,5-Triazine-2,4,6-triamine, *N,N′,N″*-tris [4-[(1,4-dimethylpentyl)amino]phenyl] (TMPPD), *N*-isopropyl-*N′*-phenylenediamine (IPPD), and the TMQ. The free radicals of an amine antioxidant could capture and combine with the active peroxide produced by the oxidation reaction of rubber molecular chain growth to form stable compounds, which could slow down the aging process [18]. Amine antioxidants show a great inhibitory effect on the aging process caused by oxygen and O_3_ oxidation, thermal interactions, buckling, and copper. However, they could cause photochromism under sunlight, resulting in the discoloration of white rubber. Thus, they are unsuitable for white and light-colored rubber products [19].

### 2.2. Phenolic Antioxidants

Phenolic antioxidants could be divided into alkylene phenolic, substituted monobasic phenolic, polybasic phenolic, and sulfurized disubstituted phenolic antioxidants [20]. Typical phenolic antioxidant products include 2,2′-methylenebis (6-tert-butyl-4-methyl-phenol) (antioxidant 2246), 2,6-di-tert-butyl-4-methylphenol (BHT (264)), and styrenated phenol (antioxidant SP). Among them, antioxidant 2246 has a good performance to protect rubber from aging caused by heat, oxygen, and metals. Because hydrogen in phenolic antioxidants can combine with the oxygen in air, their antiaging efficiency is therefore lowered compared with amine antioxidants [21,22]. By contrast, phenolic antioxidants have less effect on rubber color, and thus are widely used in light-colored rubber products [23].

### 2.3. Heterocyclic Antioxidants

Heterocyclic antioxidants are mainly used to prevent thermal and oxygen aging and could effectively prevent copper damage [24]. They are generally used in light-colored and transparent rubber products, as well as foam latex products. The commercial products of heterocyclic antioxidants mainly include 2-mercaptobenzimidazole (MB), 2-mercaptomethylbenzimidazole (MMB), 2-mercaptobenzimidazole zinc salt (MBZ), 2-mercaptomethylbenzimidazole zinc salt (MMBZ) and the benzothiazole derivatives [25]. Among them, MB and MBZ are the two most common products. MB is nontoxic but bitter, which makes it unsuitable for rubber products contacting with food, such as sanitary gloves and food container gaskets [26,27].

### 2.4. Phosphite Antioxidants

Phosphite, as a hydroperoxide-decomposing agent and a free-radical-trapping agent, plays a key role as an auxiliary antioxidant in polymer systems [28]. Phosphite antioxidants mainly include tris(nonylphenyl) phosphate (TNP), tris(1,2,2,6,6-pentamethylpiperidinyl) phosphite (GW-540), and tris(2,4-di-tert-butylphenyl) phosphite (Irgafos168). GW-540 is widely used in tires blended with styrene butadiene rubber and polybutadiene rubber. TNP is suitable for NR, SR, latex, and plastic products. As a stabilizer and antioxidant, it could endow rubber products with considerable heat resistance [29]. In addition, phosphite antioxidants have no influence on the color and luster of rubber. By the combined usage of phenols or amine antioxidants, they could effectively improve the comprehensive antiaging abilities of rubber [30].

## 3. Formation Mechanism of TPs in the Environment

**Figure 2:**
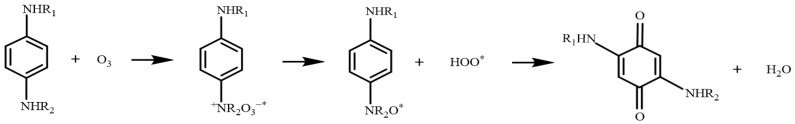
The mechanism of the PPD antioxidant ozonation (* represents radical) [34,35].

The TPs of rubber antioxidants have been observed in some studies under environmental conditions. As one of the widespread rubber antioxidants, amine antioxidants (PPDs: TMPPD, DPPD, 6PPD, and 6PPDTZ) could react with O_3_ (in parts per billion volume levels) in the environment and produce PPD-quinone [31]. A previous study clarified the early stage of oxidation reaction for various PPDs as a reaction between free radical cations and amine groups in PPDs in the presence of O_3_ [32]. The reactive mechanism between O_3_ and PPDs is given in Figure 2. O_2_^•−^ is generated as a short-lived intermediate in O_3_ reaction, and could react quickly with PPDs in the aqueous solution. This was supported by the observation of a new band of TPs in the spectrum when O_3_ passed through the aqueous solution of PPD (TMPPD), which revealed the reaction between the amine groups in PPDs and the free radical cations produced by O_3_ under oxidation conditions. In addition, some other amine antioxidants with similar structures of amine groups were also supposed to have similar reactions with O_3_, which was suggested to be the main mechanism producing toxic quinone TPs of PPDs [33].

Reaction stages between O_3_ and PPDs were observed using electron spin resonance coupled with electronic absorption spectroscopy. It was found that the respective radical cations PPD^+•^ were given by PPDs (6PPD and 77PD) after the reaction with O_3_ through the electron transfer mechanism, whereas not all PPDs ozonation could give radical cation (e.g., PPDTZ), which might suggest the different reactive efficiencies among PPDs in the solution with O_3_. Thereafter, one-electron oxidation of PPD^+•^ (6PPD^+•^ and 77PD^+•^) led to formation of the PPD-quinone [34,35]. Diverse types of TPs were produced theoretically attributed to the variety of PPD^+•^. In addition, the precursor of PPD-quinone (quinonediimine) could mix with the other part of the solution or react with other PPDs, resulting in generation of various TPs from the same antioxidants [36,37].

It was observed that nineteen 6PPD-derived TPs could be detected through ozonation of pure 6PPD [38]. These TPs could also be detected by ozonation of TWP rubbers, indicating that a variety of TPs might be generated from the same antioxidant [38]. One field study indicated that 6PPD-quinone had the highest level in dust when the atmospheric O_3_ reached the highest concentration in Tokyo, Japan [39], which further supported the assumption of O_3_ oxidative degradation. Another study investigated the formation of 32 probable 6PPD-derived TPs, and they were proven to be generated either directly from 6PPD or indirectly through 6PPD-quinone as an intermediate [40]. In addition, the water cycle has been supposed to be one of the factors that accelerates the transformation of TPs, and the concentrations of 12 Hexamethoxymethylmelamine (HMMM)-derived TPs increased in water after the sewage treatment [41]. Although the formation mechanism of PPD-derived TPs was more discussed in previous studies, the ubiquitous formation of the TPs from other antioxidants could be predicted owing to the strong oxidation effect of O_2_^•−^ produced by O_3_. However, the complete mechanism of their transformation in the environment still needs further investigation.

## 4. Toxic Effects of Antioxidants and TPs

It has been reported that short-term exposure to amine antioxidants, especially PPDs, may slightly irritate eyes and skin, whereas long-term repeated exposure may lead to skin allergy. Moreover, 6PPD even could cause angio-neurotic edema, methemoglobinemia, acute tubular necrosis, and hepatotoxicity after ingestion [42,43]. For phenolic antioxidants, the antioxidant 2246 has been suspected to be an occupational hepatotoxin and could cause dose-dependent toxicity to the liver [44]. It might also be a reproductive toxin and damage male reproductive function. It has also been proven that heterocyclic antioxidants, such as MB and MMB, can cause lethargy, ataxia, coma, changes in liver weight and hypothyroidism in rats, and strong irritation to skin and eyes [45,46]. Moreover, Irgafos168, a typical phosphite antioxidant, showed cytotoxicity and might influence the viable cell density even at much low levels [47,48]. These results indicated the evidently toxic effects of antioxidants, which required special attention to their biological health impact and environmental risks.

More and more studies have confirmed the toxicity of some PPDs and their TPs. A recent study has proved the adverse impact of 6PPD on embryonic development of zebrafish. The LC_50_ of 6PPD on zebrafish was 2.2 mg/L [49]. Another study also revealed that zebrafish embryos exhibited the phenomena of embryo solidification, spinal curvature, lack of somite formation, and weak heartbeat after 96 h of 6PPD exposure [50]. In addition, zebrafish presented decreased hatchability, reduced body length, movement disorders, and even deformity, which might be related to abnormity in the hormone levels and expression of related genes after the exposure to 6PPD [51]. Besides fish, 6PPD in water bodies has a negative impact on the growth of the rotifer (*Brachionus calyciflorus*), a common freshwater herbivore, although the toxic effect of 6PPD on crustaceans could be negligible [52]. IPPD also showed adverse effects on zebrafish. After exposure to IPPD at the concentrations of 0, 0.0012, 0.0120, and 0.1200 mg/L for 5 days, the hatchability of zebrafish embryos decreased, the ability of movement weakened, and the length of body reduced [53]. These results all indicated that PPDs might hinder the growth of fish.

6PPD-quinone, as one of the typical PPD-derived TPs, has been proved to have adverse effect on the various fish. Varshney et al. found the acute toxicity and abnormality in morphology, swimming behavior, heart rate, and oxygen consumption in zebrafish larvae caused by 6PPD-quinone [51]. The 24 h LC_50_ of 6PPD-quinone on zebrafish larvae was 309 µg/L, revealing the toxicity of 6PPD-quinone to fish in the early stages of life. Some other acute toxicity test with 6PPD-quinone showed abnormal swimming behaviors of medaka within 1 h [50]. It was also observed that 6PPD-quinone was toxic to rainbow and brook trout [54]. One hundred percent of brook trout died within 3 h when the concentrations of 6PPD-quinone was 4.4 μg/L, whereas all the rainbow trout died after 60 h when the concentrations was 1.4 μg/L. In addition, both species displayed symptoms with increased ventilation, wheezing, spiral rise, and imbalance before death. Mahoney et al. also found that 6PPD-quinone might disrupt mitochondrial respiration of rainbow trout gill cells (RTgill-W1). The oxygen consumption rate was increased rapidly in 15 min in RTgill-W1 cell after being exposed to 5–80 μg/L 6PPD-quinone, implying the tissue-specific toxicity of 6PPD-quinone [55]. 6PPD-quinone was also observed to be highly toxic to silver salmon with an LC_50_ of 0.79 ± 0.16 μg/L, which is almost three orders of magnitude lower than that of 6PPD [7]. This suggested that the TPs of antioxidants might be more toxic than their parent compounds in the environment. Further study on coho salmon with a commercial standard of 6PPD-quinone showed that the updated LC_50_ value (95 ng/L) was 8.3-fold lower than that previously reported [56]. As for the toxic symptoms caused by the 6PPD-quinone, silver salmon showed spinning and exhibited a loss of balance and fish-surface cracking within 90 min, and died within 5 h after being exposed to 20 μg/L 6PPD-quinone. Therefore, prevalent 6PPD and 6PPD-quinone in freshwater bodies has been estimated to be the main reason for the acute death of silver salmon in Seattle, WA, USA [7,57,58].

The toxic effect of other antioxidants (heterocyclic antioxidants) was also realized recently. Kawasaki et al. found that MB, a typical heterocyclic antioxidant, could be an environmental endocrine disrupter [45]. When the rats were exposed to 50 mg/kg of MB, their body weights increased and their food consumption decreased, although there were no acute toxic signs for all rats. In addition, MMB, another important heterocyclic antioxidant, might have the same antithyroid toxicity as MB. The weights of lung, liver, and kidney, and the serum cholesterol and phospholipids were significantly increased in the dose groups of 20 and 100 mg/kg, and the thyroid weight even increased 1.8 times at a dose of 100 mg/kg [59]. These studies indicated that both MB and MMB showed an antithyroid toxicity. However, adverse effects of their TPs on the biota need further verification.

## 5. Environmental Occurrence and Potential Impact of Antioxidants and TPs

The concentrations of various antioxidants and their TPs in water, dust, and air are summarized in Table 1. The rubber antioxidant might be released into the environment during the life cycle of tires, and especially the wear of automobile tires [60,61]. The antioxidants and TPs carried by TWPs were transported into river or soil through runoff [57,62,63], while those in the smaller tire particles could even enter the atmosphere during the life-cycle of automobile tires. Previous studies have revealed that only 12% of tire particles finally reached the surface water, whereas up to 67% entered soil, and the rest entered the atmospheric environment [64,65].

TWPs are the key carrier for PPD-derived TPs in the migration from rubber products to water. Storm events are considered an important factor influencing the transport of antioxidants and TPs, their concentrations increased more than 40 times during storms in the surface water samples collected from a southwestern tributary of the Brisbane River (Australia) [66]. Moreover, the concentrations of HMMM in surface water were evidently increased in the rainfall season and the snow-melting period, which also suggested the road runoff as a main pathway for antioxidant transport into urban surface water [67]. Therefore, stormwater overflows into sewers, runoff, or even commercial sources such as car washes may all affect the distribution of antioxidants and TPs in water [68]. Furthermore, it was observed that 6PPD-quinone in surface water was ubiquitous (<0.05–24 ng/L) at five urban centers in Queensland, Australia [58]. 6PPD-quinone was also prevalent in surface water and standing road water in Michigan, USA [69], and even in source waters collected in Guangzhou, China [70]. In addition, 1,3-diphenylguanidine (DPG), 6PPD-quinone, HMMM, and TPs of HMMM were all detected in municipal wastewater and drinking water from a water treatment plant in southern Ontario, Canada [68]. These studies revealed a ubiquity of antioxidants and their TPs in water. Some studies have revealed that the different adsorption capacity in suspended particles may affect variations of concentrations in water (Table 1). For instance, the excellent adsorption capacity for IPPD makes it dominant in suspended particles in water [70]. Moreover, the fate of antioxidants and TPs has been demonstrated by the temporal variation of DPG, HMMM, 6PPD-quinone, and HMMM TPs in two rivers in Toronto, Canada [71], where their concentrations are obviously higher during wet events than those during dry weather.

PPDs and their TPs were also detected in air. In the six typical megacities of China, the contaminations of PM_2.5_-bound PPDs were mostly in the pg/m^3^ level (Table 1). The detection rate of 6PPD-quinone was 81% in the urban PM_2.5_ [72]. In addition, five PPDs (IPPD, DPPD, CPPD, 6PPD, DTPD) and their TPs (IPPD-quinone, DPPD-quinone, CPPD-quinone, 6PPD-quinone, DTPD-quinone) were detected in the air samples collected in Hong Kong, China. The concentrations of DPPD-quinone had a particularly high proportion (75.9%) in the total PPD-quinones in air particles [73]. Eight PPDs (6PPD, 7PPD, DPPD, IPPD, 77PD, CPPD, DTPD, DNPD) and six PPD-quinones (IPPD-quinone, CPPD-quinone, 6PPD-quinone, 77PD-quinone, DPPD-quinone, DTPD-quinone) were also detectable in PM_2.5_ collected from three sites located in Taiyuan and Guangzhou, China [31]. This indicated that atmospheric PPDs and PPD-quinones are ubiquitous in China. It was observed that PPD-derived quinones in urban PM_2.5_ samples exhibited significant positive correlations with their parent compounds and atmospheric concentrations of O_3_, suggesting that PPD-derived quinones in the air should not be overlooked [73,74]. It was notable that traffic emission was significantly related to the distribution of PPDs and PPD-quinones in air, and their concentrations in the roadside air samples were obviously higher (Table 1) [31,74]. This may not only reveal the traffic impact on the occurrence of antioxidants and their TPs in air, but also further confirm that road runoff is a main pathway of antioxidants into the urban surface water.

Although there are no studies on antioxidants and their TPs in soil, prevailing 6PPD and 6PPD-quinone were observed in the dust of main roads and residential roads in Tokyo, Japan [39] and different roads, vehicles, underground parking lots, and houses in Guangzhou, China [75]. The concentration of 6PPD-quinone along the high-traffic-flow roads was higher than that in the residential areas, indicating the impact of traffic flow on their release. By contrast, 77PD, another typical PPD, had a relatively high proportion in indoor dust, owing to its prevailing application in indoor electrical appliances and the rubber coating of wires [75].

The ubiquity of antioxidants and their TPs in the environment implied the potential exposure risk to the biota. A simulation study showed that these coarse particles containing 6PPD-quinone could exist in the alveolar area of the workers’ airways with deposition efficiency of 89–91% [76], which might alert the inhalation risk of 6PPD-quinone and other atmospheric TPs on the public. In addition, the size of some TWPs was similar to that of secondary microplastics [77,78], which resulted in TWPs exposure to aquatic organisms, as well as the antioxidants and TPs [79,80]. This might produce a synergistic toxic effect and cause increased harm to biological individuals [81,82,83]. In this aspect, the antioxidants and TPs accumulated in aquatic organisms may subsequently enter the human body through the food chain. The study of the bioaccumulation and biomagnification of these pollutants were scarce, and requires specific concern in the future studies.

**Table 1:** The levels of antioxidants and TPs in the different environments.

Environmental Medium	Compound	Sampling Location	Concentrations	References
Water (µg/L)	DPG	Urban streams (Canada)	0.76 ± 0.05	[71]
		WWTP Discharge (Canada)	0.06 ± 0.01	
		Seattle-area waterways (America)	0.02	[84]
		Regional center (Queensland, Australia)	<0.1	[58,66]
		Brisbane (Queensland, Australia)	0.05–1.08	
	6PPD-Q	The influent of WWTP treating wastewater in the snow-melt day (Leipzig, Germany)	0.11 ± 0.04	[40]
		Urban streams (Canada)	0.72 ± 0.26	[71]
		Near WWTP Discharge (Canada)	0.05 ± 0.02	
		Surface water (Michigan, America)	<0.04	[69]
		Standing road water (Michigan, America)	0.05–0.66	
		Regional center (Queensland, Australia)	<0.02	[58,66]
		Brisbane (Queensland, Australia)	<0.09	
		Urban river in the Don River (Canada)	2.30 ± 0.05	[85]
		Runoff water (Hong Kong, China)	0.21–2.43	[73]
	HMMM	Urban streams (Canada)	2.26 ± 0.34	[71]
		Surface water in the Don River and Highland Creek (Canada)	>1	[67]
		Regional center (Queensland, Australia)	<0.29	[58,66]
		Brisbane (Queensland, Australia)	0.01–0.20	
	HMMM	German rivers (Germany)	0.01–0.88	[86]
		Water treatment plants, influent (Southern Ontario, Canada)	<0.01–0.03	[68]
		Water treatment plants, effluent (Southern Ontario, Canada)	0.02–0.11	
	HMMM TPs	Urban streams (Canada)	<11.2	[71]
Dust (ng/g)	6PPD	Road (Tokyo, Japan)	45–1175	[39]
		Road (Guangzhou, China)	4.1–238	[75]
		Parking lot (Guangzhou, China)	13.5–429	
		Vehicle (Guangzhou, China)	5.0–41.9	
		House (Guangzhou, China)	n.d.^a^–6.1	
		Roadside soils (Hong Kong, China)	31.4–831	[73]
		Indoor dust (Beijing)	n.d.–0.28	[87]
		Playground dust (Beijing)	n.d.–0.69	
	6PPD-Q	Road (Tokyo, Japan)	870–8520	[39]
		Road (Guangzhou, China)	32.2–80.9	[75]
		Parking lot (Guangzhou, China)	5.7–277	
		Vehicle (Guangzhou, China)	17.9–146	
		House (Guangzhou, China)	n.d.–0.4	
		Roadside soils (Hong Kong, China)	9.50–936	[73]
		E-waste recycling workshops (south China)	375	[88]
	77PD	Road (Guangzhou, China)	n.d.–38.5	[75]
		Parking lot (Guangzhou, China)	n.d.–29.1	
		Vehicle (Guangzhou, China)	n.d.–9.6	
		House (Guangzhou, China)	n.d.–77.6	
	DNPD	Road (Guangzhou, China)	1.5–35.9	[75]
		Parking lot (Guangzhou, China)	n.d.–28.9	
		Vehicle (Guangzhou, China)	1.9–29.5	
		House (Guangzhou, China)	n.d.–137	
	CPPD	Road (Guangzhou, China)	3.4–190	[75]
		Parking lot (Guangzhou, China)	5.8–540	
		Vehicle (Guangzhou, China)	5.2–66.8	
		House (Guangzhou, China)	n.d.–0.4	
		Roadside soils (Hong Kong, China)	0.73–15.4	[73]
	DPPD	Road (Guangzhou, China)	5.8–126	[75]
		Parking lot (Guangzhou, China)	16.4–217	
		Vehicle (Guangzhou, China)	n.d.–55.3	
		House (Guangzhou, China)	n.d.–27.0	
		Roadside soils (Hong Kong, China)	3.63–84.4	[73]
		Indoor dust (Beijing)	n.d.–22.2	[87]
		Playground dust in Beijing	n.d.–22.6	
	IPPD	Road (Guangzhou, China)	n.d.–321	[75]
		Parking lot (Guangzhou, China)	n.d.–237	
		Vehicle (Guangzhou, China)	n.d.–575	
		House (Guangzhou, China)	n.d.–41.5	
		Roadside soils (Hong Kong, China)	0.66–24.5	[73]
		E-waste recycling workshops (south China)	363	[88]
Air (pg/m^3^)	IPPD	Hong Kong Baptist University (Hong Kong, China)	0.44–2.73	[73]
		Shanxi University (Taiyuan, China)	0.3–8.3	[72]
		Zhengzhou University (Zhengzhou, China)	0.3–50	
		Fudan University (Shanghai, China)	0.3–104	
		Jiangsu Provincial Center for Disease Control and Prevention (Nanjing, China)	0.8–4.7	
		Government of Hangzhou Binjiang District (Hangzhou, China)	0.4–2.4	
		Guangdong University of Technology (Guangzhou, China)	0.2–5.7	
	DPPD	Hong Kong Baptist University (Hong Kong, China)	n.d.–0.70	[73]
		Shanxi University (Taiyuan, China)	0.1–8.2	[72]
		Zhengzhou University (Zhengzhou, China)	0.1–1.5	
		Fudan University (Shanghai, China)	0.1–5.6	
		Jiangsu Provincial Center for Disease Control and Prevention (Nanjing, China)	0.1–13	
		Government of Hangzhou Binjiang District (Hangzhou, China)	0.1–5.8	[72]
		Guangdong University of Technology (Guangzhou, China)	0.1–1	
	CPPD	Hong Kong Baptist University (Hong Kong, China)	n.d.–0.74	[73]
		Shanxi University (Taiyuan, China)	0.5–14	[72]
		Zhengzhou University (Zhengzhou, China)	0.4–4.2	
		Fudan University (Shanghai, China)	0.4–21	
		Jiangsu Provincial Center for Disease Control and Prevention (Nanjing, China)	0.3–1.2	
		Government of Hangzhou Binjiang District (Hangzhou, China)	0.4–3.0	
		Guangdong University of Technology (Guangzhou, China)	0.1–5.1	
	6PPD	Hong Kong Baptist University (Hong Kong, China)	0.82–6.30	[73]
		Shanxi University (Taiyuan, China)	0.02–487	[72]
		Zhengzhou University (Zhengzhou, China)	1.2–109	
		Fudan University (Shanghai, China)	0.5–135	
		Jiangsu Provincial Center for Disease Control and Prevention (Nanjing, China)	0.4–75	
		Government of Hangzhou Binjiang District (Hangzhou, China)	0.1–6.0	
		Guangdong University of Technology (Guangzhou, China)	0.3–10	
	DNPD	Shanxi University (Taiyuan, China)	0.5–14	[72]
		Zhengzhou University (Zhengzhou, China)	0.6–7.1	
		Fudan University (Shanghai, China)	0.5–108	
		Jiangsu Provincial Center for Disease Control and Prevention (Nanjing, China)	0.3–4.7	
		Government of Hangzhou Binjiang District (Hangzhou, China)	1.4–9.9	[72]
		Guangdong University of Technology (Guangzhou, China)	0.5–5.5	
	77PD	Shanxi University (Taiyuan, China)	0.2–7052	[72]
		Zhengzhou University (Zhengzhou, China)	0.5–231	
		Fudan University (Shanghai, China)	0.05–967	
		Jiangsu Provincial Center for Disease Control and Prevention (Nanjing, China)	0.1–84	
		Government of Hangzhou Binjiang District (Hangzhou, China)	0.5–93	
		Guangdong University of Technology (Guangzhou, China)	0.1–693	
	6PPD-Q	Hong Kong Baptist University (Hong Kong, China)	0.54–13.8	[73]

n.d. = not detected.

## 6. Conclusions and Future Perspectives

Antioxidants are widely used to improve the performance of rubber, and their production, especially 6PPD, is annually maintained at a high level [60]. Amine antioxidants and TPs have been generally detected in the environment, especially in water, air, and dust, indicating that they can be transported through the atmosphere and rivers. Their ubiquity in the environment may result in biota exposure risk of amine antioxidants and TPs. However, the environmental risks caused by these compounds are not well understood. In a landmark study [7], scientists confirmed that the TP (6PPD-quinone) of amine antioxidant (6PPD) showed high toxicity and caused the acute death of silver salmon in Seattle, WA, USA. More and more studies have revealed the toxic effects of the TPs of antioxidants, which has recently raised great concern about the environmental impact of rubber antioxidants and their TPs. Concerning the research status on their environmental occurrence and impact, the following research aspects may be prioritized in the near future:

(1) Since the environmental incident by 6PPD-quinone raised great concern recently, several studies have been conducted to reveal the environmental occurrence and risk of antioxidants and their TPs. Therefore, suspect and non-target screening of these chemicals should be carried out in various environmental and biota matrices [89]. In addition, the production and impact of TPs from photo-, thermo-, and bio-degradation should also be considered. Notably, antioxidants are generally used to prevent rapid aging of rubber, which suggests a high environmental persistence of these chemicals. The study on their environmental behaviors should be strengthened, especially the environmental impacts of those beyond 6PPD in various environments, including polar regions.

(2) The toxic effects of rubber antioxidants and their TPs may be evaluated via computational toxicology combined with traditional toxicological methods, including studies of short-term and sub-lethal exposure effects, the potential for mortality, mechanism of effect, and interaction with environmental variables. Computational toxicology could enhance understanding of toxicity mechanisms and predict toxic effects, using mathematics, informatics, and computer models [90]. These are more suitable for the initial evaluation of toxic effects compared to traditional methods, especially in the case of more chemicals being introduced into the environment [91,92,93].

(3) Biological effects of antioxidants and TPs should also be put on the agenda, including bioavailability, bioaccumulation, and biomagnification through the food chain. Since the studies on toxic effects of antioxidants and TPs are limited, and only several publications reported their observations in fish, their biological effects on both the terrestrial and aquatic organisms are still unknown. Moreover, the biodegradation and metabolization of these pollutants may also occur, which means that the possible degradation products should be paid more attention due to their unknown toxicity.

## Data Availability

The study did not report any data.
